# Theory driven psychological therapy for persecutory delusions: trajectories of patient outcomes

**DOI:** 10.1017/S0033291724002113

**Published:** 2024-11

**Authors:** Lucy Jenner, Mollie Payne, Felicity Waite, Helen Beckwith, Rowan Diamond, Louise Isham, Nicola Collett, Richard Emsley, Daniel Freeman

**Affiliations:** 1 Institute of Psychiatry, Psychology & Neuroscience, King's College London, London, UK; 2 Department of Experimental Psychology, Oxford Cognitive Approaches to Psychosis, University of Oxford, Oxford, UK; 3 Oxford Health NHS Foundation Trust, Oxford, UK; 4 Aneurin Bevan University Health Board, Wales, UK

**Keywords:** cognitive therapy, delusions, outcome trajectories, persecutory, psychosis

## Abstract

**Background:**

We aimed to identify the common types of outcome trajectories for patients with psychosis who take up specialist psychological therapy for persecutory delusions. Knowing the different potential responses to therapy can inform expectations. Further, determining predictors of different outcomes may help in understanding who may benefit.

**Methods:**

We analyzed delusion conviction data from 767 therapy sessions with 64 patients with persistent persecutory delusions (held with at least 60% conviction) who received a six-month psychological intervention (Feeling Safe) during a clinical trial. Latent class trajectory analysis was conducted to identify groups with distinct outcome profiles. The trajectories were validated against independent assessments, including a longer-term follow-up six months after the end of therapy. We also tested potential predictors of the trajectories.

**Results:**

There were four outcome trajectories: (1) Very high delusion conviction/Little improvement (*n* = 14, 25%), (2) Very high delusion conviction/Large improvement (*n* = 9, 16%), (3) High delusion conviction/Moderate improvement (*n* = 17, 31%) and (4) High delusion conviction/Large improvement (*n* = 15, 27%). The groups did not differ in initial overall delusion severity. The trajectories were consistent with the independent assessments and sustained over time. Three factors predicted trajectories: persecutory delusion conviction, therapy expectations, and positive beliefs about other people.

**Conclusions:**

There are variable responses to psychological therapy for persecutory delusions. Patients with very high delusion conviction can have excellent responses to therapy, though this may take a little longer to observe and such high conviction reduces the likelihood of positive responses. A trajectory approach requires testing in larger datasets but may prove highly informative.

## Introduction

The main reports of randomized clinical trials provide the average outcome for a patient allocated to an intervention compared to a patient allocated to a control condition. For example, we reported that there was a large additional improvement in delusion conviction (Cohen's *d* = 0.86) for patients allocated to receive a new specialist cognitive therapy for persecutory delusions (Feeling Safe) compared to patients who were allocated to receive a befriending intervention (Freeman et al., 2021a). However, the reality is that variable responses to the cognitive therapy were observed during the trial. This will be true of all psychological therapies for psychosis but to our knowledge there has been no reporting of different types of responses. Repeated data collected in therapy sessions has the potential to identify longitudinal outcome trajectories (Emsley & Dunn, 2010). Analyzing therapy session-by-session data has proven highly beneficial in improving the understanding and treatment of common mental health disorders (Clark et al., 2018; Skelton et al., 2023). In this paper we use session-by-session ratings of degree of conviction in persecutory delusions collected in the Feeling Safe trial to understand how patients respond over the course of therapy, who therapy may work well for, and where there is need for improvement.

A person-centered approach – classifying individuals into distinct groups based on individual response patterns over time - has the potential to be clinically informative and help direct how to improve an intervention. For example, it could give clinicians and patients a better description of what may happen with provision of an intervention. It may help identify who is likely to benefit, or not benefit, from a specific treatment approach. It could test certain clinical folklore, such as that patients with very high certainty in their delusions will not be suitable for psychological therapy. And by identifying predictors of poorer outcomes, it could provide indications of how to improve an intervention (Banerjee et al., 2023).

Latent class trajectory analysis – a probabilistic method of unsupervised clustering - provides a way to identify distinct subgroups of individuals with similar patterns of change over time (Van De Schoot, Sijbrandij, Winter, Depaoli, & Vermunt, 2016). It involves modelling longitudinal data using a mixture model framework, where the latent classes are inferred based on observed data collected at multiple time points (Sinha, Calfee, & Delucchi, 2020). It has been used to study responses to psychological therapies for a number of clinical conditions such as depression and anxiety (Altmann et al., 2020; Skelton et al., 2023; Terrill et al., 2022). It has also been used to study trajectories of response to antipsychotic medication in patients diagnosed with psychosis and patients at ultra-high risk of psychosis (Austin et al., 2015; Chen et al., 2013; Hartmann et al., 2020; Stauffer et al., 2011). Typically two to four change patterns are identified. One group is normally characterized by an absence of response to treatment, and the remaining groups are characterized by improvement that varies in size and timing of occurrence.

We had two main objectives in the current study. Our first objective was to use session-by-session therapy data to identify the different trajectories of patients with persistent persecutory delusions who received our six-month, theory driven, Feeling Safe therapy. We also sought to validate these trajectories by use of the independent blinded rater trial assessments. Our second objective was to identify potential predictors of the different response trajectories.

## Methods

### Participants

The data were collected from sixty-four participants who received Feeling Safe in the main randomized controlled trial (Freeman et al., 2021a). The sample size was therefore predetermined. The trial received approval from an NHS Research Ethics Committee (South Central – Oxford B Research Ethics Committee; ref 15/SC/0508). Participant inclusion criteria were: aged 16+ years old; a primary clinical diagnosis of non-affective psychosis; and experiencing a persecutory delusion (defined by Freeman and Garety (2000) that had persisted for at least three months and held with at least 60% conviction. Participants were excluded if they were receiving another psychological therapy, had an insufficient comprehension of English, had a current primary diagnosis of substance or personality disorder, were being treated in forensic mental health services, had an organic syndrome, or had a learning disability.

### The intervention

Feeling Safe is a theory-driven cognitive therapy for persecutory delusions (Freeman et al., 2016; Freeman, 2016). It is an individual therapy, provided by clinical psychologists weekly over six-months. The underlying therapy design principles have been described recently (Freeman, 2024). An average of 19 therapy sessions was attended by patients in the trial. Patients work through with the therapist a number of modules, each comprising a set of booklets, targeting a cause of persecutory delusions. For example, there are modules targeting worry, negative self-beliefs, disrupted sleep, voices, and defense behaviors. The overall aim is to help the person build up new memories of safety that counteract the persecutory fears. In each session there is measurement of degree of conviction in the delusion. A number of sessions are held outside to inform learning and for practical reasons ratings would be less likely to be taken for these sessions.

### Outcomes

In each Feeling Safe therapy session the therapist asked the patient to rate their conviction in the persecutory belief (e.g. ‘My neighbours are trying to physically harm me’) on a scale of 0–100%, where 0% is ‘Do not believe’ and 100% is ‘Absolutely certain’. These session-by-session ratings have not previously been analyzed.

All of the outcome measures used in the original Feeling Safe analysis (Freeman et al., 2021a) were also available. These data were collected by blinded research assistants at three time points: baseline, six months (post-treatment), and twelve months (follow up). Baseline outcome measures included in the final analysis and current report were conviction and severity in the persecutory delusion rated within the Psychotic Symptoms Rating Scale - Delusions (PSYRATS) (Haddock, McCarron, Tarrier, & Faragher, 1999), expectations of the effectiveness of therapy assessed with the Credibility/Expectancy Questionnaire (Devilly & Borkovec, 2000), positive beliefs about other people assessed using the Brief Core Schema Scale (BCSS) (Fowler et al., 2006) and belief flexibility measured by the possibility of being mistaken rated 0–100% (Waller et al., 2015).

### Statistical analysis

Analyses were conducted in the statistical software STATA version 18 (StataCorp). Descriptive data analysis was carried out to report the feasibility of collecting within-therapy session data and to evaluate missingness to determine the validity of further statistical analyses.

A basic latent class trajectory model was run to determine whether there were sub-groups of people, or latent classes, who responded to Feeling Safe in a similar way. We modelled a series of basic latent growth trajectory analyses, starting with a non-mixture one-class solution (Sinha et al., 2020). We then compared models with increasing numbers of latent classes until the optimal solution was found based on the lowest value Bayesian Information Criteria (BIC), predicted probabilities of group assignment, and expert opinion. Predicted probabilities of belonging to the relevant class were examined for each class model. A threshold of 0.85 for predicted probabilities for the relevant class implies confidence in class assignment, and therefore a robust model. To optimize the latent class trajectory model, additional constraints that made theoretical sense were included to improve the power of the model. We constrained the error variance of the persecutory delusion rating at each session to be consistent over all time points. We compared this basic model with a model that also constrained the mean change of persecutory threat belief at each session to change at the same rate within each class. The optimal model reveals how many latent groups of people there are who respond similarly to the specialized therapy.

To further optimize the latent class trajectory model, we compared a series of latent growth trajectory models with different conditions on classes and probabilities. The basic model, with unconditional trajectory classes and unconditional class probabilities (Model 1), involved a two-step procedure of identifying classes and evaluating their association with baseline covariates. Associations with all baseline assessment measures from the Feeling Safe trial were evaluated with separate multinomial logistic regression models, using the largest class as the reference category. Baseline assessment measures that were associated with class individually, with a *p*-value of less than 0.05, were then included in the adjusted model. We conducted a series of multinomial logistic models and compared model fit across models with different numbers of predictors, optimizing for the least number of predictors that had good explanatory power.

Model 1 was compared with an unconditional trajectory classes and conditional class probabilities model (Model 2), where we allowed the baseline covariates to predict the class. These models were then compared to a conditional trajectory class and unconditional probabilities model (Model 3), where the covariates predict persecutory delusion conviction at each session and classes assignment is determined by the remaining variation in persecutory delusion conviction. To determine the best performing model they were compared based on how they aligned with psychological theory, the predicted probabilities of relevant class assignments, and the values of the BIC.

The best-performing model enabled us to classify individuals into one of the identified latent classes to evaluate the patterns in outcome trajectories. We compared the mean and standard deviations of variables across the four latent classes descriptively to investigate potential differences across the latent classes. This included baseline predictors of trajectory class, measures of therapy, and other baseline measures.

To validate the latent trajectory classes, we examined the persecutory delusion ratings collected by the blinded trial research assistants at baseline (pre-therapy), 6 months (post-therapy), and 12 months (follow up).

## Results

1066 therapy sessions were included in the analysis, which were from the first therapy session up to the nineteenth therapy session from 64 patients randomized to the Feeling Safe treatment. The persecutory delusion belief conviction rating was collected in 767 (72%) of these therapy sessions. Only nine people had more than half of the persecutory delusion conviction ratings missing. There was no pattern to the missingness of data, with each participant having a distinct missing data pattern. Data could therefore be assumed to be missing at random (MAR).

### Identifying trajectories of outcomes and predicting latent classes

Basic latent class analysis revealed that the four-class model best explained the trajectories of patients as it had the lowest BIC value (Table 1, step 1), and sufficiently high predicted probabilities of belonging to the relevant class, with 1.0 for Class 1, 0.93 for Class 2, and 0.97 for Class 3 and 4. Adding constraints to the four-class basic latent class model, Model 1, improved the fit and therefore this model was taken forward.

**Table 1.** Latent class trajectory model characteristics including number of patients within each class, predicted probabilities per class, Akiake Information Criterion (AIC), and Bayesian Information Criterion (BIC)

	Model	n	Number and predicted probability per class	AIC	BIC
**Step 1**	One Class Model	63	Class 1	7391	7433
	Two Class Model	63	Class 1– 39 (0.99)	7044	7130
			Class 2– 24 (0.96)		
	Three Class Model	63	Class 1– 31 (0.98)	6825	6953
			Class 2– 16 (0.99)		
			Class 3– 16 (0.99)		
	**Four Class Model**	**63**	**Class 1– 8** (**1.00)**	**6732**	**6903**
			**Class 2– 22** (**0.93)**		
			**Class 3– 16** (**0.97)**		
			**Class 4– 17** (**0.97)**		
	Five Class Model	63	Class 1– 7 (1.00)	6727	6942
			Class 2– 11 (0.97)		
			Class 3– 14 (0.95)		
			Class 4– 16 (0.97)		
			Class 5– 15 (1.00)		
**Step 2** **(four class models with constraints)**	Model 1: Non-mixture four-class solution, unconditional trajectory classes and unconditional class probabilities	63	Class 1– 16 (0.97)	6714	6742
			Class 2– 9 (0.97)		
			Class 3– 20 (0.92)		
			Class 4– 18 (0.97)		
	**Model 2*:* Unconditional trajectory classes and conditional class probabilities**	**55**	**Class 1– 14** (**0.98)**	**5653**	**5698**
			**Class 2– 9** (**0.97)**		
			**Class 3– 17** (**0.97)**		
			**Class 4– 15** (**1.00)**		
	Model 3*:* Conditional trajectory classes and unconditional probabilities	55	Class 1– 14 (0.96)	5683	5715
			Class 2– 14 (0.97)		
			Class 3– 14 (0.98)		
			Class 4– 15 (0.88)		

Separate multinomial logistic regression analyses with all baseline assessment measures from the Feeling Safe trial found significant associations (*p*-value of less that 0.05) between the likelihood of belonging to different classes and nine baseline variables. These were baseline delusion conviction, therapy expectancy, positive beliefs about other people, negative beliefs about other people (Fowler et al., 2006), persecutory ideation (Freeman et al., 2021b), anhedonia (Gard, Kring, Gard, Horan, & Green, 2007), belief flexibility, psychological well-being (Tennant et al., 2007), and vulnerability ratings (online Supplementary materials, Table S1). There were no significant associations between class membership and the other baseline assessment measures or socio-demographic characteristics measured in the Feeling Safe trial.

We included the nine baseline measures that had a significant association in a series of multinomial logistic models and optimized for the lowest number of predictors with good model fit. Baseline conviction, therapy expectancy, and positive beliefs about others were the best three predictors of class membership that were taken forward in Models 2 and 3. Model 2, the unconditional trajectory classes and conditional class probabilities model, includes these covariates within the model and was a better fitting model compared to Model 1, with a lower BIC of 5698 (Table 1, step 2). Model 3, the conditional trajectory classes and unconditional probabilities model, had a BIC of 5715, also lower than Model 1 but higher than Model 2. Model 2 was therefore chosen as the best performing model as it had the lowest BIC, highest predicted probabilities, and made clinical and theoretical sense.

Model 2 incorporated the three baseline factors found to predict class membership. The coefficients in the final multinomial logistic indicate the strongest predictors for each class compared to a reference class, Class 3, High conviction/Moderate improvement (Table 2). The likelihood of following the trajectory of Class 1, Very high conviction/Little improvement, was significantly predicted by holding lower positive beliefs about others and high baseline delusion conviction compared to Class 3. Lower levels of positive beliefs about other people also significantly predicted the likelihood of belonging to Class 2, Very high conviction/Large improvement, compared to Class 3. Higher expectations for the effectiveness of therapy are the strongest predictor of the likelihood of belonging to Class 4, high conviction/Large improvement compared to Class 3.

**Table 2.** Model 2: final multinomial logistic regression model with three significant predictors

Model 2: Reference class is Class 3, High conviction/Moderate improvement	Coefficient	Std. err.	*p value*	Lower CI	Upper CI
Class 1: Very high conviction/Little improvement (v. Class 3)					
Conviction	0.29	0.11	0.01	0.08	0.50
Expectancy	0.22	0.14	0.12	−0.05	0.49
Positive beliefs about others	−0.54	0.16	0.00	−0.85	−0.23
Class 2: Very high conviction/Large improvement (v. Class 3)					
Conviction	0.08	0.05	0.14	−0.03	0.19
Expectancy	0.15	0.11	0.16	−0.06	0.37
Positive beliefs about others	−0.36	0.13	0.01	−0.63	−0.10
Class 4: High conviction/Large improvement (v. Class 3)					
Conviction	−0.04	0.04	0.26	−0.12	0.03
Expectancy	0.23	0.09	0.01	0.06	0.40
Positive beliefs about others	−0.19	0.11	0.08	−0.41	0.02

### Characterizing the latent class trajectories of persecutory delusion conviction

**Figure 1.**
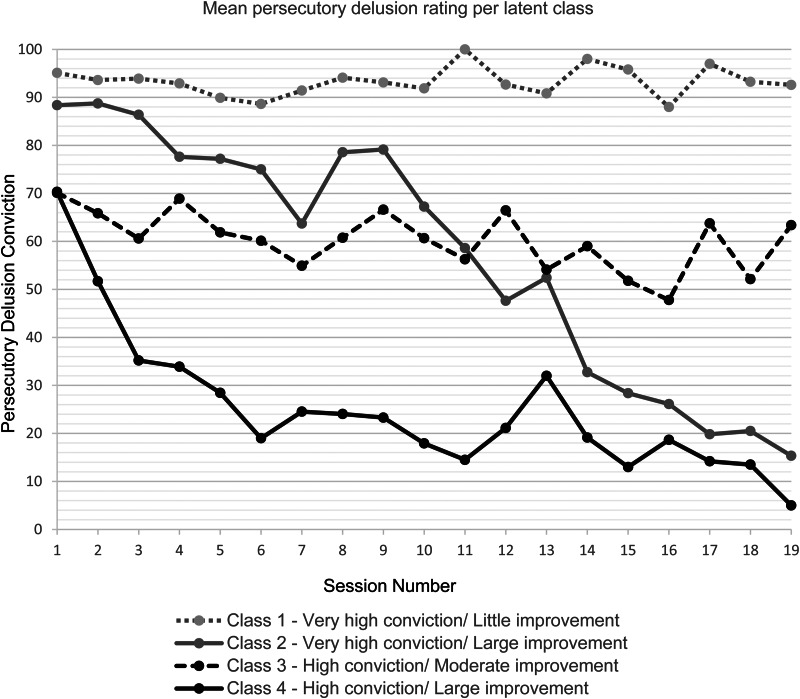
Mean persecutory delusion conviction rating per session across patients for each latent trajectory class identified within Model 2.

**Figure 2.**
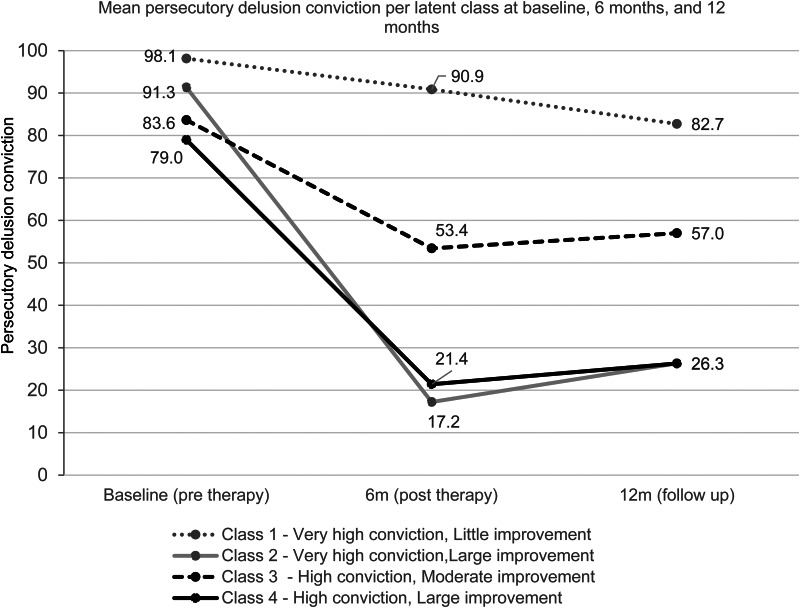
Mean persecutory delusion conviction per latent class in Model 2 at baseline (pre therapy), 6-month (post therapy) and 12-month (follow up) assessment time points recorded by research assistants.

The trajectories of patients’ persecutory delusion conviction over the course of 19 therapy sessions for each of the four latent classes are shown in Fig. 1. The labelling of the trajectories principally considers the patterns observed across the trial's independent assessments (shown in Fig. 2). There are four sub-groups of patients: (1) Very high conviction/Little improvement (*n* = 14, 25%), (2) Very high conviction/Large improvement (*n* = 9, 16%), (3) High conviction/Moderate improvement (*n* = 17, 31%), and (4) High conviction/Large improvement (*n* = 15, 27%). Descriptive statistics for each of the four latent classes are shown in Table 3. Some patients had fewer than 19 therapy sessions, therefore over the course of therapy the number of patients in each class reduces (online Supplementary materials, Table S2).

**Table 3.** Descriptive statistics of socio-demographic factors, baseline outcome measures, and therapy measures across the latent classes

		1: Very high conviction/Little improvement (*n* = 14)	2: Very high conviction/Large improvement (*n* = 9)	3: High conviction/Moderate improvement (*n* = 17)	4: High conviction/Large improvement (*n* = 15)
Predictors of trajectory class	Mean baseline conviction (s.d.)	98.1 (3.7)	91.3 (9.1)	83.6 (12.2)	79.0 (11.1)
		Range: 90–100	Range: 75–100	Range: 60–100	Range: 60–100
	Mean therapy expectancy (s.d.)	18.2 (5.5)	19.3 (4.0)	16.5 (6.6)	21.9 (4.1)
		Range: 7.5–25.4	Range: 12.4–24.6	Range: 7.2–27	Range: 13.2–26.0
	Mean positive beliefs about others (s.d.)	4.9 (4.1)	7.1 (4.8)	12.2 (3.5)	10.5 (4.9)
		Range: 0–12	Range: 1–17	Range: 6–18	Range: 1–20
Therapy measures	Mean number of sessions (s.d.)	18.5 (8.8)	21.8 (5.7)	21.3 (6.7)	15.5 (4.7)
		Range: 3–39	Range: 14–31	Range: 4–33	Range: 8–23
	Mean no. of sessions *in vivo* (s.d.)	3.9 (3.7)	5.1 (4.2)	8.7 (6.8)	6.3 (4.0)
		Range: 0–10	Range: 2–13	Range: 0–28	Range: 0–14
	Mean % of sessions *in vivo*	21%	23%	41%	41%
	Mean number of therapy modules (s.d.)	2.4 (1.0)	3.1 (1.0)	2.9 (0.8)	2.5 (0.9)
		Range: 0–4	Range: 1–5	Range: 1–4	Range:1–4
Baseline measures	Mean PSYRATS total (s.d.)	19.0 (2.4)	18.9 (2.8)	18.1 (1.9)	18.0 (2.1)
		Range: 15–23	Range: 14–23	Range: 14–22	Range: 15–22
	Mean belief flexibility (s.d.)	4.6 (13.3)	15.3 (16.7)	23.7 (14.9)	30.9 (28.6)
		Range: 0–50	Range: 0–50	Range: 0–52	Range: 0–100
Demographics					
Mean age (s.d.)		47.9 (10.0)	38.8 (14.6)	39.8 (13.9)	37.4 (9.5)
Gender (n)	Male	6 (42.9%)	4 (44.4%)	11 (64.7%)	9 (60.0%)
	Female	8 (57.1%)	5 (55.6%)	6 (35.3%)	6 (40.0%)
Ethnicity (n)	White	10 (71.4%)	7 (77.8%)	16 (94.1%)	11 (73.3%)
	Black Caribbean	1 (7.1%)	1 (11.1%)		1 (6.7%)
	Black African		1 (11.1%)	1 (5.9%)	
	Black other	1 (7.1%)			
	Indian	1 (7.1%)			1 (6.7%)
	Pakistani	1 (7.1%)			1 (6.7%)
	Chinese				
	Other				1 (6.7%)
Marital status (n)	Single	8 (57.1%)	7 (77.8%)	16 (94.1%)	8 (53.3%)
	Married	5 (35.7%)	2 (22.2%)	1 (5.9%)	5 (33.3%)
	Divorced	1 (7.1%)	0 (0.0%)	0 (0.0%)	2 (13.3%)
Employment (n)	Unemployed	9 (64.3%)	7 (77.8%)	16 (94.1%)	12 (80.0%)
	Other	5 (35.7%)	2 (22.2%)	1 (5.9%)	3 (20.0%)

### Class 1: Very high conviction/little improvement

Class 1 had the highest mean baseline delusion conviction (95.1%, s.d. = 8.8) and showed little improvement during the course of therapy. Persecutory delusion conviction remained very high, above 90%, after 19 sessions. Class 1 had the lowest mean positive beliefs about others compared to the other classes.

### Class 2: Very high conviction/large improvement

Class 2 had very high persecutory delusion conviction at baseline (88.4%; s.d. = 10.1). The mean conviction rating was slightly lower than Class 1 but a number of patients, as in Class 1, had 100% conviction in their delusion before starting therapy. During the course of therapy patients experienced large improvements, with a mean reduction from around 90% conviction to 15%. Progress was somewhat slow for the first half of therapy, with conviction still at 80% after nine therapy sessions. After this point, delusion conviction reduced substantially at each therapy session.

### Class 3: High conviction/moderate improvement

Baseline delusion conviction was high for Class 3 before therapy, 70.3% conviction (s.d. = 20.4), but lower than Classes 1 and 2. Overall the trend was less clear with the most improvement seen by session 16 when mean delusion conviction fell below 50%. Class 3 had the highest positive beliefs about others but the lowest expectations of therapeutic benefit.

### Class 4: High conviction/large improvement

Class 4 also had high conviction (70.1%, s.d. = 21.2) but responded very well to psychological therapy. After three sessions the mean persecutory delusion rating had already reduced by almost 40 percentage points and continued to reduce further as sessions progressed. At baseline, Class 4 had the highest expectations of the therapy effectiveness, followed by Class 2, the two groups that gained large improvements from therapy. Compared to Class 2 the improvement in persecutory delusion conviction occurred more rapidly.

The four latent classes differed in the mean number of Feeling Safe therapy sessions they received and the proportion of those that took place outside of their home or clinic room. The High conviction/Large improvement group had the fewest therapy sessions (mean = 15.5, s.d. = 4.7), on average 5 sessions fewer than those with High conviction/Moderate improvement (mean = 21.8, s.d. = 5.7). The Very high conviction/Little improvement group received fewer sessions (mean = 18.5, s.d. = 8.8) than the Very high conviction/Large improvement group (mean = 21.3, s.d. = 6.7), but still received close to the overall average number of 19 sessions. The two sub-groups with very high conviction had approximately 20% of sessions outside the therapy room, compared to 40% for those with high conviction.

Overall delusion severity as assessed by the PSYRATS was similar across the four classes. There was however a difference between the groups at baseline in belief flexibility, the possibility of being mistaken rated on a scale of 0–100%. In the Very high conviction/Little improvement group this was lower than in the Very high conviction/Large improvement group, suggesting that belief flexibility was associated with better outcome. Belief flexibility was highest in the High conviction/Large improvement group, and slightly lower in the High conviction/Moderate improvement group.

### Validation against the independent trial assessments

The persecutory delusion ratings collected by trial research assistants at baseline (before therapy), 6 months (post therapy) and 12 months (follow up) per latent trajectory class are shown in Fig. 2, and were used to inform the naming of the four classes.

Overall, 43% of patients (*n* = 24) who received the specialized psychological therapy experienced large improvements during therapy, reducing persecutory delusion conviction by approximately 60–70 percentage points. For a further 30% of patients (*n* = 17) there were moderate improvements (reduction of delusion conviction by approximately 30 percentage points). The improvements were all maintained at the 12-month follow-up. The remaining 27% of patients (*n* = 14) experienced little improvement with the psychological therapy.

Patients in Classes 1 and 2 had very high persecutory delusion conviction at the start of therapy, over 90%, but responded very differently to therapy. Class 2 responded very well to therapy, with a 74 percentage point reduction in persecutory delusion conviction during therapy, and the effects were maintained 6 months after the end of therapy. Class 1 did not improve substantially within the time they were receiving therapy, with persecutory delusion conviction still very high at the end of therapy. After therapy, there was a decrease by a further eight percentage points to 83%, but this remains very high.

Patients in Classes 3 and 4 started with high persecutory delusion conviction (84%, Class 3 and 79%, Class 4). Class 4 responded very well to the psychological therapy, showing large reductions in persecutory delusion conviction that were maintained six months after the end of therapy. Class 3 did not respond as well to the therapy as Class 4, but still saw a substantial reduction in persecutory delusion conviction of 30 percentage points. This reduction was maintained after the end of therapy.

## Discussion

This study is the first to examine the outcome trajectories of people diagnosed with psychosis receiving psychological therapy. It is clear that patients do not respond in the same way. We identified four different outcome trajectory classes. Two groups experienced large improvements with therapy in their persecutory delusions, with one group starting from very high conviction in their delusion and one group starting from high conviction in their delusion. Another group experienced moderate improvements in the delusions and one group showed no benefit of receiving therapy. These outcomes, found from session-by-session therapy data, were validated by the main trial assessments. These four groups did not differ in initial overall delusion severity as assessed by the most used global delusion scale. However there were intriguing indicators of initial differences between the groups. Persecutory delusion conviction, therapy expectancy, and beliefs about other people were found to predict group membership. Overall, the results can provide clinicians and patients with more realistic descriptions of what may happen with therapy and point to how the intervention might be improved. More broadly, this study shows the feasibility and value of collecting session-by-session therapy data.

The finding of four classes is similar to recent studies of trajectories of anxiety and depression symptoms during therapy for common mental health disorders (Murphy & Smith 2018; Skelton et al., 2023). When four classes are found it often includes a group with gradual improvement, fast improvement, initially mild with some improvement, and a minimal change group. In our study around 40% of patients experienced large improvements in persecutory delusion conviction (16% gradual improvement from very high conviction and 27% fast improvement from high conviction). A further 30% experienced moderate improvements from a high conviction and 30% showed little improvement from a very high conviction. If a patient started with a very high or a high delusion conviction level, the likelihood of gaining large improvements from the therapy was comparable, 40% (high) compared to 47% (very high). The difference was that for individuals who did not gain large improvements with therapy, those with high conviction saw moderate improvements, whereas those with very high conviction did not seem to have any improvement. Very high delusion conviction therefore reduced the likelihood of a treatment response.

It is important to note that patients with very high conviction in their persecutory delusion could have very large improvements with the Feeling Safe intervention even though baseline delusion conviction was a predictor of treatment response. This included patients who were 100% sure in their belief. However, this improvement may take a little longer to observe compared to patients with less high delusion conviction that experienced large improvements. If delusion conviction was very high (over 90%) it is likely more sessions are required before improvement in the delusion occurs. If persecutory delusion conviction was not very high at the start of therapy then improvement was quicker, and these individuals had fewer therapy sessions. These patients experienced significant improvements even within the first three sessions. Clinically it may therefore be important to persist with the individuals with very high conviction, not to stop therapy prematurely, even if there has not been improvement in the first eight sessions.

Therapy expectancy and positive beliefs about other people were found to predict outcome trajectories. Interestingly, having a higher level of pre-existing positive beliefs about other people may differentiate patients with very high delusion conviction who do respond to the intervention compared to patients with very high conviction who do not respond to the intervention. Specifically targeting positive beliefs about others has rarely been done in people with psychosis but one non-clinical experimental study has highlighted its potential for reducing paranoia (Brown, Waite, Rovira, Nickless, & Freeman, 2020). For patients with less high delusion conviction, it may be positive expectations of therapy that differentiate whether people respond well to the therapy or not. This supports evidence that building expectations of change is important (Constantino, Visla, Coyne, & Boswell, 2018). These findings are potential clues about psychological processes, moderated by level of belief conviction, that may aid recovery in persecutory delusions. It suggests factors that could be targeted in newer iterations of the Feeling Safe treatment. For example, it would be worthwhile to assess expectancy of treatment outcome systematically at the start of the intervention. If expectations of change are low, then discussing with the person why there may be pessimism about the potential for change and how Feeling Safe is designed to work could be included. It may also be helpful to include numerous different examples of patient experiences of the intervention.

The key limitation of this study was the pre-determined sample size. The sample size limits the statistical power to detect potentially rarer classes and factors predictive of class membership (Nylund-Gibson & Choi, 2018). There were also missing data, including in the baseline predictors, which further reduced the sample size used in the final model. The data were found to be missing at random. However, there may be unobserved patterns to missingness, for example, the therapist who provided treatment or the time point within the research trial. Participants with greater levels of missing data are likely to have had less clear class membership. It is also the case that the focus was on one outcome – persecutory delusion conviction – and we do not know about potential trajectories for other important outcomes (Greenwood et al., 2010). It is both a limitation and a strength that all the data were collected from one clinical trial, at one location, with a small number of clinical psychologists providing the intervention. The limitation is the potential generalizability of the findings, especially as there were few patients from ethnic minority groups. The strength is that since many variables were tightly controlled the statistical power to identify consistent patterns in outcome trajectories will have been greater. Despite the theoretically stable class solution found, it would be valuable to confirm the stability and build upon the findings by replication in a larger dataset (Nylund-Gibson & Choi, 2018; Weller, Bowen, & Faubert, 2020). Session-by-session therapy outcome data are rarely collected for patients with psychosis (Jensen-Doss et al., 2018). This trial produced an opportunity to examine such data and validate it against independent assessments collected outside of therapy. The clinically plausible trajectories, and indications of predictors of outcomes, show the potential benefits of taking an individual trajectories approach. If treatment services routinely collect such data (Fornells-Ambrojo et al., 2017) – as has happened in UK psychological therapy services for common mental health conditions (Clark et al., 2018) – then there is considerably more learning that could be made.

## Supporting information

Jenner et al. supplementary material Jenner et al. supplementary material

## Supplementary material

The supplementary material for this article can be found at https://doi.org/10.1017/S0033291724002113.
