# Supplementary material for: Theory driven psychological therapy for persecutory delusions: trajectories of patient outcomes
Source: Psychol Med. 2024 Nov 18;54(15):4173–81. doi: 10.1017/S0033291724002113 (PMC11650162; doi:10.1017/S0033291724002113)
Supplement: Jenner et al. supplementary material [file S0033291724002113sup001.docx]

**Theory driven psychological therapy for persecutory delusions: trajectories of patient outcomes**

Lucy Jenner^*1^, Mollie Payne^1^, Felicity Waite ^2,3^, Helen Beckwith ^2,3^, Rowan Diamond ^2^, Louise Isham^2,3^, Nicola Collett ^4^, Richard Emsley^1^ and Daniel Freeman ^2,3^

^1^ Institute of Psychiatry, Psychology & Neuroscience, Kings College London, London, UK

2 Oxford Cognitive Approaches to Psychosis, Department of Experimental Psychology, University of Oxford, Oxford, UK

3 Oxford Health NHS Foundation Trust, Oxford, UK

^4^Aneurin Bevan University Health Board, Wales, UK

**Supplementary materials:**

**Table S1 Multinomial regression output of significant results of Model 1 with the largest class as reference, Class 3.** Belief flexibility is measured by the possibility of being mistaken rated 0-100%. Persecutory ideation is measured using the Revised Green et al Paranoia Thoughts Scale part b. Negative beliefs about other people is measured using the Brief Core Schema Scales). Vulnerability (“I feel vulnerable”) is measured on 0-100 visual analogue scale. Psychological wellbeing is measured using the Warwick-Edinburgh Mental Wellbeing Scale. Anhedonia is measured using the anticipatory scale of the Temporal Experience of Pleasure Scale.

| **Class** | **Baseline variable** | **Coefficient** | **Std. error** | **p-value** | **Lower CI** | **Upper CI** |
| --- | --- | --- | --- | --- | --- | --- |
| Class 1 vs 3 | Belief flexibility | -0.078 | 0.030 | 0.010 | -0.138 | -0.019 |
| Class 1 vs 3 | Persecutory ideation | 0.159 | 0.056 | 0.005 | 0.048 | 0.269 |
| Class 1 vs 3 | Delusion conviction | 0.218 | 0.073 | 0.003 | 0.076 | 0.361 |
| Class 1 vs 3 | Negative beliefs about other people | 0.243 | 0.089 | 0.007 | 0.068 | 0.418 |
| Class 1 vs 3 | Positive beliefs about other people | -0.358 | 0.105 | 0.001 | -0.563 | -0.152 |
|  |  |  |  |  |  |  |
| Class 2 vs 3 | Persecutory ideation | 0.198 | 0.075 | 0.008 | 0.051 | 0.344 |
| Class 2 vs 3 | Vulnerability | 0.077 | 0.031 | 0.013 | 0.016 | 0.137 |
|  |  |  |  |  |  |  |
| Class 4 vs 3 | Therapy expectancy | 0.142 | 0.071 | 0.044 | 0.004 | 0.281 |
| Class 4 vs 3 | Anhedonia | 0.065 | 0.033 | 0.050 | 0.000 | 0.131 |
| Class 4 vs 3 | Psychological well-being | 0.093 | 0.047 | 0.049 | 0.000 | 0.185 |

**Table S2: Number of people (n) and percentage of missing data (% missing) in each therapy session per latent class from final Model 2.**

|  |  | **Total** | **1: Very high conviction / Little improvement (n=14)** | | **2: Very high conviction / Large improvement (n=9)** | | **3: High conviction/ Moderate improvement (n=17)** | | **4: High conviction/ Large improvement (n=15)** | |
| --- | --- | --- | --- | --- | --- | --- | --- | --- | --- | --- |
|  | n | % missing | n | % missing | n | % missing | n | % missing | n | % missing |
| Session 1 | 55 | 13% | 14 | 29% | 9 | 11% | 17 | 0% | 15 | 13% |
| Session 2 | 55 | 18% | 14 | 7% | 9 | 11% | 17 | 18% | 15 | 33% |
| Session 3 | 55 | 22% | 14 | 29% | 9 | 11% | 17 | 12% | 15 | 33% |
| Session 4 | 54 | 26% | 13 | 15% | 9 | 11% | 17 | 35% | 15 | 33% |
| Session 5 | 53 | 32% | 13 | 23% | 9 | 44% | 16 | 50% | 15 | 13% |
| Session 6 | 53 | 21% | 13 | 15% | 9 | 22% | 16 | 13% | 15 | 33% |
| Session 7 | 53 | 26% | 13 | 46% | 9 | 22% | 16 | 25% | 15 | 13% |
| Session 8 | 52 | 25% | 12 | 25% | 9 | 22% | 16 | 19% | 15 | 33% |
| Session 9 | 51 | 26% | 12 | 33% | 9 | 22% | 16 | 19% | 14 | 29% |
| Session 10 | 51 | 25% | 12 | 33% | 9 | 11% | 16 | 44% | 14 | 7% |
| Session 11 | 51 | 43% | 12 | 75% | 9 | 44% | 16 | 31% | 14 | 29% |
| Session 12 | 50 | 36% | 12 | 50% | 9 | 11% | 16 | 31% | 13 | 46% |
| Session 13 | 48 | 40% | 11 | 45% | 9 | 22% | 16 | 44% | 12 | 42% |
| Session 14 | 46 | 35% | 11 | 55% | 9 | 11% | 16 | 38% | 10 | 30% |
| Session 15 | 44 | 34% | 10 | 50% | 9 | 11% | 16 | 44% | 9 | 22% |
| Session 16 | 42 | 43% | 10 | 50% | 8 | 0% | 16 | 69% | 8 | 25% |
| Session 17 | 39 | 44% | 10 | 80% | 8 | 25% | 14 | 36% | 7 | 29% |
| Session 18 | 37 | 43% | 9 | 56% | 8 | 25% | 14 | 50% | 6 | 33% |
| Session 19 | 30 | 20% | 6 | 17% | 7 | 0% | 13 | 38% | 4 | 0% |
